# Upon a Method of Treatment Preventive of Puerperal Fever

**Published:** 1857-05

**Authors:** M. Piedagnel


*Upon a Method of Treatment Preventive of Puerperal Fever.* By M. Piedagnel. (From the *Gazette Medicale.*)

M. Piedagnel communicated to the Academy of Sciences, Paris, in its sitting of Nov. 24, 1856, the following note upon a method of Treatment Preventive of Puerperal Fever:—During an epidemic of puerperal fever, at Paris, lying-in women were distributed through the various hospitals, and a certain number were received into the wards at Hotel Dieu, under the charge of M. Piedagnel. Conscious of the uncertainty of medication against this disease, M. Piedagnel thought it might not be impossible to prevent its occurrence, and forthwith endeavored to discover the means.

Knowing that quinine had often been employed with advantage in this disease, and that it prevented the access of pernicious intermittent fever, a disease usually more severe than puerperal fever, and recalling that during the cholera of 1853–54 he had obtained undoubted preventive results from its use; knowing also that iron, which has a positive action upon all the economy, has also been employed with advantage against puerperal fever, it seemed that by associating them good results might accrue from their administration. But as puerperal fever ordinarily commences suddenly, and is not always preceded by any partial alteration, he thought the administration of these medicaments, which could not produce any injurious result, might be made before the appearance of the disease, when its irruption was feared.

The patients he received were well watched, and kept carefully clean. The windows of the wards were kept open almost all the time, even at night, when the weather would permit; fire was kept day and night in the stoves, so as to produce currents of air, and the treatment used was as follows:—

As soon as a woman entered the wards to lie-in, or if she had been delivered, she took two pills, each containing about one and a-half grains of quinine and fifteen grains of sub-carbonate of iron, and in the evening the same quantity; and as long as she remained in the hospital she took morning and evening the same dose, drinking linden-flower water and a bottle of Spa water. All the functions were watched and preserved as much as possible in their physiological integrity. This was the treatment in simple cases, but in those in whom the signs of the fever had become developed, the dose of the medicament was increased progressively each day as high as 5, 10, and 15 grains of the sulphate of quinine, and of a ʒj to a ʒiss of the iron. As soon as the symptoms became milder, the amount of the medicaments were reduced.

Of 94 women delivered under his care, one only died of puerperal fever contracted in his wards.—*Amer. Medical Monthly.*

